# Complete genome sequence of *Anaerotignum* sp. strain MB30-C6, isolated from sewage sludge of the wastewater treatment plant at a steel factory

**DOI:** 10.1128/mra.00078-24

**Published:** 2024-03-19

**Authors:** Fu Feng, Chih-Hung Wu, Chao-Jen Shih, Yen-Chi Wu, Shu-Jung Lai, Yi-Ting You, Sheng-Chung Chen

**Affiliations:** 1 College of Environment and Safety Engineering, Fuzhou University, Fuzhou City, Fujian, China; 2 School of Resources and Chemical Engineering, Sanming University, Sanming City, Fujian, China; 3 Fujian Provincial Key Laboratory of Resources and Environmental Monitoring and Sustainable Management and Utilization, Sanming University, Sanming, Fujian, China; 4 Bioresource Collection and Research Center, Food Industry Research and Development Institute, Hsinchu, Taiwan, China; 5 Graduate Institute of Biomedical Sciences, China Medical University, Taichung City, Taiwan, China; 6 Research Center for Cancer Biology, China Medical University, Taichung City, Taiwan, China; 7 Department of Life Sciences, National Chung Hsing University, Taichung City, Taiwan, China; DOE Joint Genome Institute, Berkeley, California, USA

**Keywords:** sewage sludge, *Anaerotignum*, steel factory, wastewater treatment, rolling-tube technique

## Abstract

We report the complete genome sequence of *Anaerotignum* sp. strain MB30-C6, which was isolated from the dehydrated sludge collected at the wastewater treatment plant of Sanming Steel Co. Ltd. in Fujian, China. The resulting genome of strain MB30-C6 is a single contig of 3,104,838 bp with 39.49% GC content.

## ANNOUNCEMENT

**Fig 1:**
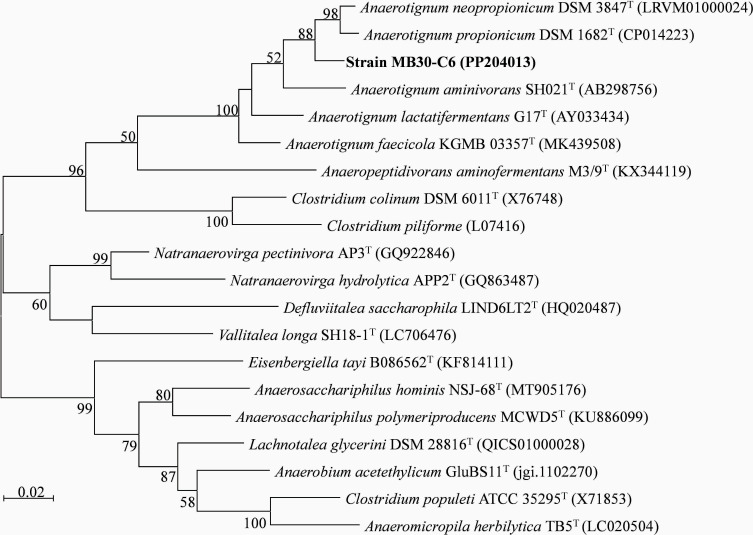
Phylogenetic analysis of 16S rRNA gene sequences showing the relationship between strain MB30-C6 and related taxa. The tree was reconstructed using the MEGA11 software package (5) based on near-full-length sequences and using the maximum likelihood method. The multiple sequence alignment was carried out using ClustalW (6). Bootstrap values at the nodes were expressed as percentages of 1,000 replications. GenBank or JGI accession numbers are shown in parentheses. Bar, 0.02 substitutions per nucleotide position.

The potential biodegrade of key pollutants in steel industry wastewater, such as oils, phenols, and cyanides, was explored using a culture-dependent method. Strain MB30-C6 was isolated from the dehydrated sludge obtained from the wastewater treatment plant of Sanming Steel Co. Ltd, located in Fujian, China, on 25 June 2021. Genome mining of strain MB30-C6 identified four lipase-related genes. Approximately 1 mL of the sludge was inoculated into the anaerobic modified DSM924 medium (without acetate and formate) and incubated at room temperature (~ 25°C) for 2 weeks. To purify and identify strain MB30-C6, a combination of methods was employed, including serial dilution, rolling-tube technique (1), and the bacterial 16S rRNA gene cloning and sequencing, as detailed in our previous study (2). Based on 16S-based ID analysis at the EZBioCloud website (3), strain MB30-C6 exhibited the highest similarity (97.32%) to *Anaerotignum neopropionicum* DSM 3847^T^ (4). Furthermore, a phylogenetic tree analysis of the 16S rRNA gene, conducted using MEGA11 (5) for strain MB30-C6 and related taxa, indicated that strain MB30-C6 is situated within the clade of genus *Anaerotignum* (Fig. 1). The genome of strain MB30-C6 was sequenced for species delineation and comparative genomic analysis.

Cells of strain MB30-C6 were cultivated in modified DSM 924 medium at 35°C for 2 days. Genomic DNA was extracted from a cell pellet collected from 1 L culture using the NucleoBond HMW DNA kit (Macherey-Nagel, Germany), following the manufacturer’s instructions. The genome was sequenced at the Sangon Biotech (Shanghai) Co., Ltd. utilizing the DNBSEQ-T7 platform (MGI Tech Co., Ltd.) and the MinION sequencer (Oxford Nanopore Technology).

For the DNBSEQ-T7 platform, genomic DNA fragments with an average size of approximately 300 bp were utilized to construct a paired-end DNA library with 150 bp inserts. The library preparation was executed using the Hieff NGS MaxUp II DNA Library Prep Kit for Illumina from Yeasen Biotechnology (Shanghai) Co., Ltd. Subsequently, the constructed library underwent sequencing using MGISEQ-2000RS High-throughput Sequencing Set (PE150 format), yielding a total of 10,730,800 reads with an average read length of 150 bp. These reads were then subjected to trimming using Trimmomatic v0.36 (7). In the case of the MinION sequencer, the Covaris g-TUBE sheared DNA fragments from the same DNA extract underwent end-repair, 3′ adenylation, and ligation to adaptors pre-loaded with motor proteins. Subsequently, fragments larger than 1 Kb, purified with Agencourt AMPure XP Beads (Beckman, A63881), were subjected to single-molecule nanopore DNA sequencing using the Multiplex Ligation Sequencing Kit (SQK-MLK111.96-XL) on a MinION Flow Cell (R9.4.1). Basecalling was performed using Guppy version 6.5.6 with the default model, resulting in a total of 283,391 reads and *N*_50_ of 6,328 bp. Trimming of the reads was carried out using Porechop v0.2.4 (8), and filtering was executed with NanoFilt v2.8.0 (9). The MinION reads were assembled using Canu v2.2 (10). Gap filling and sequence correction were performed by integrating DNBSEQ-T7 reads using GapFiller (11) and PrInSeS-G (12), respectively. This resulted in a circularized contig containing 3,104,838 bp with 39.49% GC content. Gene predictions and annotations were performed using NCBI Prokaryotic Genome Annotation Pipeline (PGAP) v6.5 (13). Default parameters were used for all bioinformatics analyses. Table 1 presents the basic characteristics of both the strain and the genome.

**TABLE 1:** Basic characteristics of *Aminobacterium* sp. strain MB30-C6 based on MIGS (Minimum Information for Genome Sequence) recommendation and general information on its genome

Item	Description
MIGS data	
NCBI BioProject	PRJNA1006636
NCBI BioSample	SAMN37044124
GenBank accession number	CP133078
Geographic location	Sewage sludge of Wastewater Treatment Plant of Sanming Steel Co. Ltd., Sanming City, Fujian, China
Latitude and longitude	26.2649 N, 117.6245 E
Collection date	2021.06.25
Sequencing platforms	DNBSEQ-T7 and Oxford Nanopore MinION
Assembly method	Canu v. 2.2
Coverage	858.0x
Finishing strategy	Complete
General features of strain	
Classification	Domain: *Bacteria* Phylum: *Bacillota* Class: *Clostridia* Order: *Eubacteriales* Family: *Lachnospiraceae* Genus: *Anaerotignum*
Gram stain	positive
Cell shape	rod-shaped
Relationship to oxygen	Anaerobic
Optimal growth temperature	35°C
Optimal growth pH	7.0
Optimal growth NaCl concentration	1.0%
Genomic features	
Size (bp)	3,104,838 bp
GC content (%)	39.49
CDSs (with protein)	2,942
Number of tRNAs	60
Number of rRNAs	7, 6, 6 (5S, 16S, 23S)

## Data Availability

The genome sequence of strain MB30-C6 has been deposited in GenBank under accession number CP133078. The version of the genome described in this paper is the first version. The BioProject and BioSample accession numbers are PRJNA1006636 and SAMN37044124. DNBSEQ-T7 and MinIONTM raw reads were deposited in the Sequence Read Archive (SRA) under accession numbers SRR27218394 and SRR27218393, respectively. The 16S rRNA gene sequence of strain MB30-C6 has been deposited in GenBank under the accession number PP204013
